# Establishment of a Novel Mouse Model for Atherosclerotic Vulnerable Plaque

**DOI:** 10.3389/fcvm.2021.642751

**Published:** 2021-03-16

**Authors:** Xueyu Wang, Yahong Fu, Zulong Xie, Muhua Cao, Wenbo Qu, Xiangwen Xi, Shan Zhong, Minghui Piao, Xiang Peng, Ying Jia, Lingbo Meng, Jinwei Tian

**Affiliations:** ^1^Department of Cardiology, The Second Affiliated Hospital of Harbin Medical University, Harbin, China; ^2^Key Laboratory of Myocardial Ischemia, Chinese Ministry of Education, Harbin, China; ^3^Department of Cardiology, The Second Affiliated Hospital of Chongqing Medical University, Chongqing, China

**Keywords:** atherosclerosis, vulnerable plaque, animal model, plaque rupture, Fbn1^C1039G +/–^LDLR^–/–^ mice

## Abstract

**Background and Aims:** Acute coronary syndrome (ACS) is a group of clinical syndromes characterized by rupture or erosion of atherosclerotic unstable plaques. Effective intervention for vulnerable plaques (VP) is of great significance to reduce adverse cardiovascular events.

**Methods:** Fbn1^C1039G+/−^ mice were crossbred with LDLR^−/−^ mice to obtain a novel model for atherosclerotic VP. After the mice were fed with a high-fat diet (HFD) for 12 or 24 weeks, pathological staining and immunohistochemistry analyses were employed to evaluate atherosclerotic lesions.

**Results:** Compared to control mice, Fbn1^C1039G+/−^LDLR^−/−^ mice developed more severe atherosclerotic lesions, and the positive area of oil red O staining in the aortic sinus was significantly increased after 12 weeks (21.7 ± 2.0 vs. 6.3 ± 2.1) and 24 weeks (32.6 ± 2.5 vs. 18.7 ± 2.6) on a HFD. Additional vulnerable plaque characteristics, including significantly larger necrotic cores (280 ± 19 vs. 105 ± 7), thinner fiber caps (14.0 ± 2.8 vs. 32.6 ± 2.7), apparent elastin fiber fragmentation and vessel dilation (3,010 ± 67 vs. 1,465 ± 49), a 2-fold increase in macrophage number (8.5 ± 1.0 vs. 5.0 ± 0.6), obviously decreased smooth muscle cell number (0.6 ± 0.1 vs. 2.1 ± 0.2) and an ~25% decrease in total collagen content (33.6 ± 0.3 vs. 44.9 ± 9.1) were observed in Fbn1^C1039G+/−^LDLR^−/−^ mice compared with control mice after 24 weeks. Furthermore, spontaneous plaque rupture, neovascularization, and intraplaque hemorrhage were detected in the model mouse plaque regions but not in those of the control mice.

**Conclusions:** Plaques in Fbn1^C1039G+/−^LDLR^−/−^ mice fed a HFD show many features of human advanced atherosclerotic unstable plaques. These results suggest that the Fbn1^C1039G+/−^LDLR^−/−^ mouse is a novel model for investigating the pathological and physiological mechanisms of advanced atherosclerotic unstable plaques.

## Introduction

Atherosclerosis (AS), which results from the accumulation of lipid-rich plaques within artery walls, has become one of the biggest risks to human health. Studies have demonstrated that the growth of atherosclerotic plaques has obvious stages (1). It is widely believed that in the early stage, due to endothelial dysfunction, lipids and lipid-laden macrophages accumulate in the subendothelial area, leading to the formation of foam cells (2). Subsequently, with the continuous progression of plaques and exacerbation of the inflammatory response, a large number of growth factors and inflammatory mediators are secreted by macrophages and other cells, and lipid streaks gradually emerge (3). Moreover, as smooth muscle cells (SMCs) proliferate and migrate to intimal tissue, more elastin and collagen are produced, and lipid streaks gradually change into fibrous plaques. Advanced plaques are enlarged fibrous plaques characterized by narrowed lumen areas, haemodynamic changes, thinner fibrous caps and larger necrotic cores (4). In general, pathological and imaging studies have demonstrated that there are “stable” and “vulnerable” plaques in human coronary arteries (5). Stable plaques, which are accompanied by collagen accumulation, can form a fibrous plaque without a necrotic core. In contrast, vulnerable plaques are characterized by a large necrotic core and a thinner fibrous cap and have a high risk of sudden rupture and thrombosis leading to acute coronary events (5). In recent years, although considerable progress has been made in the prevention and treatment of cardiovascular diseases, atherosclerotic plaque rupture, especially vulnerable plaque rupture, is still the principal pathogenic mechanism of ACS (6, 7). Thus, identifying the specific mechanism of plaque rupture and designing effective prevention and treatment strategies are of great significance (8).

Arteriosclerosis is reported to be a major independent risk factor for cardiovascular diseases in the macro- and microvasculature. As a principal part of extracellular matrices, elastic fibers provide resilience and deformability for arterial vessels. Abnormal changes in the structure and function of elastic fibers can lead to decreased elasticity of vessels, vessel aging and arteriosclerosis. Elastic fibers are composed of two distinct components: insoluble, homogeneous, amorphous elastin, and fibrillin-rich microfibrils (9). Fibrillin-1 (Fbn1), a primary component of microfibrils, provides an architectural scaffold for elastin deposition and crosslinking. Studies have found that mutant fibrillin-1 damages the structure of the vessel wall by rupturing the crosslinking of elastic fibers, resulting in arteriosclerosis (10, 11). Based on this result, the relationship between AS and elastin fragmentation may provide a new perspective in studies of cardiovascular diseases. In previous research, multiple models of AS have been developed with a variety of methods to study the pathological mechanisms of and interventions in atherosclerosis; these methods include using lipid metabolism regulation, physical damage, drug action, and gene knockout techniques to induce atherosclerotic plaque formation. Among these models, apoprotein E-deficient mice (ApoE^−/−^) and low-density lipoprotein (LDL) receptor-deficient mice (LDLR^−/−^) have been widely used (12). However, in terms of pathology, these models of atherosclerosis represent some but not all characteristics of human ruptured plaques (13–15). Due to the limitations of human atherosclerotic plaque studies, it is urgent to develop suitable animal models of plaque rupture for basic and clinical research.

In the present study, LDLR^−/−^ mice were crossbred with Fbn1^C1039G+/−^ mice. After feeding the mice a HFD for 12 or 24 weeks, we evaluated the characteristics of plaques in LDLR^−/−^ (the control group) and Fbn1^C1039G+/−^LDLR^−/−^ (the model group) mice using multiple pathological experiments. We aimed to construct a novel atherosclerosis model for studying the pathological and physiological mechanism of the development of atherosclerotic plaques by introducing a fibrillin-1 heterozygous mutation into LDLR^−/−^ mice via homologous recombination.

## Materials and Methods

### Animals

Fbn1^C1039G+/−^ mice (C57BL/6 background, purchased from The Jackson Laboratory, Maine, USA) were crossbred with LDLR^−/−^ mice (C57BL/6 background, purchased from Beijing HuaFuKang Bioscience Co. INC, Beijing, China). According to Mendel's genetic laws, we obtained Fbn1^C1039G+/−^LDLR^−/−^ mice in the second generation. The genotype was identified by polymerase chain reaction, gene sequencing analysis, and agarose gel electrophoresis. The primer sequences were as follows: LDLR-c: CCATATGCATCCCCAGTCTT, LDLR-w: GCGATGGATACACTCAC-TGC, LDLR-m: AATCCATCTTGTTCAATGGCCGATC; C1039G-F: TTGTCCA-TGTGCTTTAAGTAGC, C1039G-R: ACAGAGGTCAGGAGATATGC. To quickly obtain enough Fbn1^C1039G+/−^LDLR^−/−^ mice, female mice were used for experiments, while males were used for breeding. Eight-week-old female Fbn1^C1039G+/−^LDLR^−/−^ mice (*n* = 10) were fed a HFD (Nanjing Junke Bioengineering Co., Ltd., # AAN01, China) containing 10% fat, 2% cholesterol, and 0.5% cholic acid. Female LDLR^−/−^ mice (*n* = 10) were used as controls. Animals were housed in a temperature-controlled room with a 12-h light/dark cycle and had free access to water and food. They were inspected daily for neurological symptoms (including head tilt, disorientation, and motor disturbances) or sudden death. Body weight was measured every 10 days in the HFD period. All experiments were approved by the Ethics Committee of the Second Affiliated Hospital of Harbin Medical University, China (2015-Yan-043).

### Tissue Collection and Processing

To evaluate the plaque characteristics of the two groups in different periods, plaque area and composition in Fbn1^C1039G+/−^LDLR^−/−^ and LDLR^−/−^ mice were analyzed after 12 or 24 weeks on a HFD. Mice were anesthetized (*n* = 10 in each group), and blood samples were taken from the retro-orbital plexus for analysis of lipid profiles by a commercially available kit (Nanjing Jiancheng Bioengineering Institute, Nanjing, China). After fixation and perfusion of the left ventricle with 4% formaldehyde (pH 7.4), tissues (heart, aorta, and brachiocephalic artery) were collected. Subsequently, the heart and arteries were fixed in 4% formaldehyde for 24 h, dehydrated overnight, and embedded in paraffin or Tissue-Tek O.C.T. (SAKURA #4583, USA). Histological analysis was performed on serial paraffin cross sections (4.5 μm) or frozen sections (6 μm).

### Histological Analysis

*En face* oil red O staining of the aorta was used to quantify the surface area occupied by atherosclerosis. Haematoxylin and eosin (H&E) staining was used to analyze plaque area and necrotic core area. The necrotic core, quantified in three 30 μm-spaced sections per artery, was defined as a hypocellular plaque cavity devoid of collagen and containing necrotic debris and cholesterol clefts. A 3,000 μm^2^ threshold was implemented to avoid counting very small H&E-negative areas. Fibrous caps were defined as elastin-rich layers covered by overlying plaque. Outward remolding meant that the vessel expanded outward and the lumen area increased. Acute plaque rupture was defined as a visible defect in the cap. Elastin and collagen contents were determined by Movat-Russell staining and Masson staining, respectively.

### Immunohistochemistry and Immunofluorescence Staining

The percentage of macrophages was determined by anti-Mac-3 immunostaining (BDPharmingen #550292, USA), the percentage of smooth muscle cells was determined by anti-α-SMA staining (Cell Signaling Technology #19245, USA), and the content of matrix metalloproteinase 2 was determined by anti-MMP-2 immunostaining (Wanleibio #WL03224, China). Secondary antibodies were species-appropriate horseradish peroxidase conjugates (Abcam, USA). 3,3′-Diaminobenzidine (KeyGEN BioTECH #KGP1045, China) was used as a chromogen. Intraplaque neovascularization and hemorrhages were examined on slides that were double stained with anti-TER119 (Biolegend #116215, America) and anti-CD31 (Abcam #ab28364, USA) antibodies.

### Statistical Analysis

Images were examined and quantified by Image-Pro Plus 6.0 software. All statistical analyses were performed with SPSS software (version 20, IBM Corporation, Armonk, NY, USA) or GraphPad Prism (version 5.0, GraphPad Software, Inc., La Jolla, CA, USA). The results are expressed as the mean ± SEM. Statistical comparisons were carried out using one-way analysis of variance (ANOVA) followed by the LSD test. The Kruskal–Wallis-test was used, followed by the Dunnett-T3 test if the variance was not equal. Differences were considered significant at *P* < 0.05 (two-sided). The numbers of mice are mentioned in the figure legends.

## Results

### Fbn1^C1039G+/–^LDLR^–/–^ Mice Exhibit Elastic Fiber Fracture and Exacerbated Atherosclerosis

**Figure 1:**
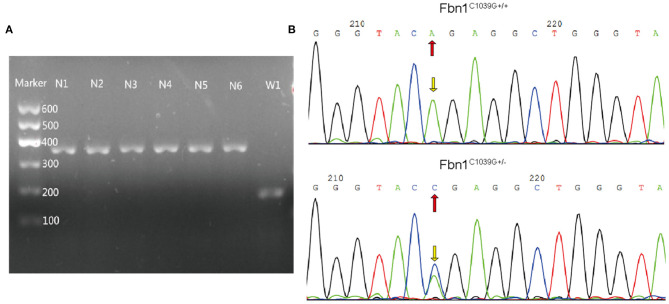
Genotype identification. **(A)** Agarose gel electrophoresis was used to identify the genotypes of LDLR^−/−^ mice. The length of the LDLR^−/−^ product was 350 bp, while that of the LDLR^+/+^ product was 167 bp. **(B)** Gene sequencing analysis showed that Fbn1^C1039G+/−^ had a bimodal waveform, with the nucleotide mutated to C, while Fbn1^C1039G+/+^ only had a single waveform, and the nucleotide was still A.

**Figure 2:**
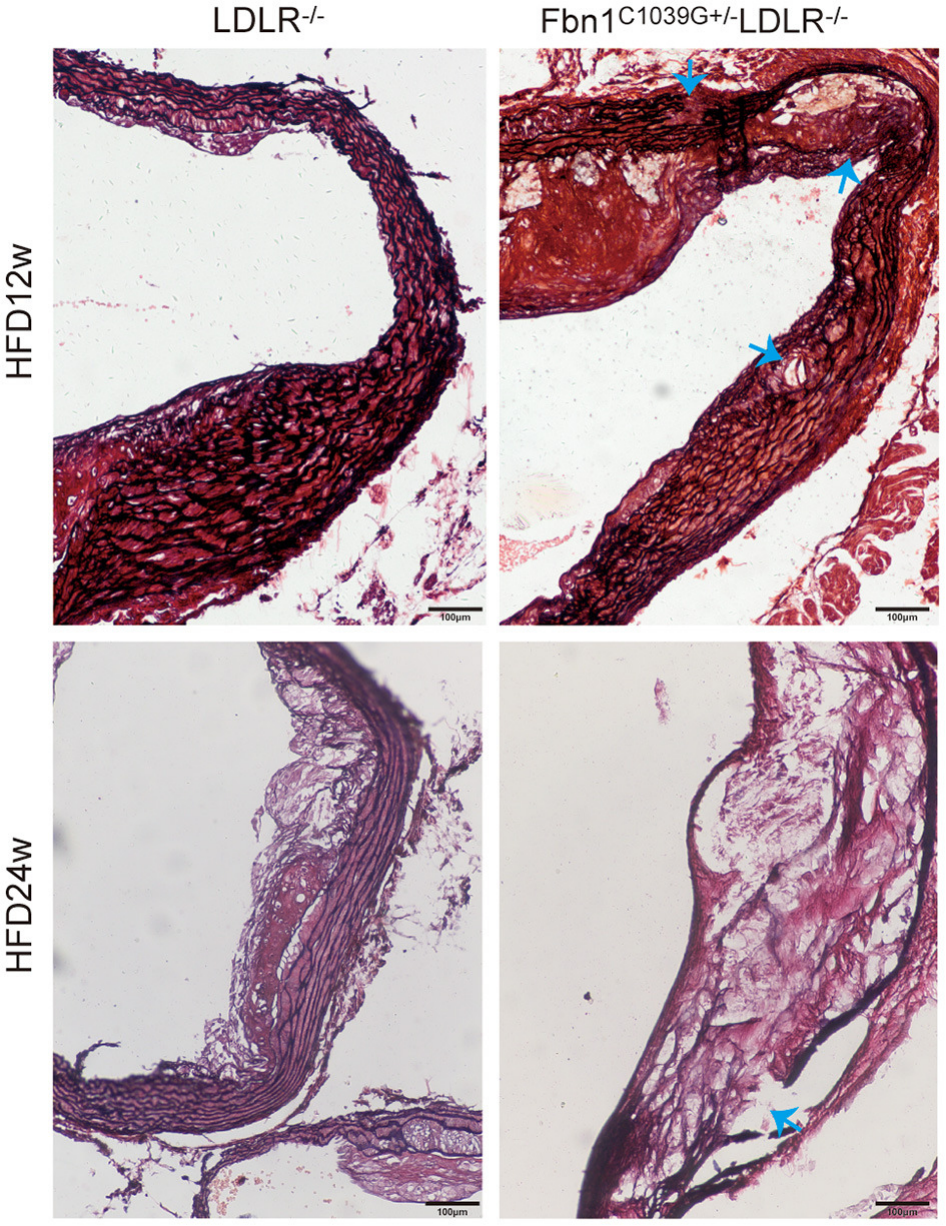
Elastin fragmentation in Fbn1^C1039G+/−^LDLR^−/−^ mice. The arrow shows Elastin fragmentation in Fbn1^C1039G+/−^LDLR^−/−^ mice after 12 and 24 w on a HFD, while elastin in LDLR^−/−^ mice was intact. Movat staining: elastin is shown in black.

To determine the effect of the mutation of the fibrillin-1 gene in LDLR^−/−^ mice, we crossbred LDLR^−/−^ mice with Fbn1^C1039G+/−^ mice to generate Fbn1^C1039G+/−^LDLR^−/−^ mice. As shown in Figures 1A,B, the genotype was identified by polymerase chain reaction, gene sequencing analysis, and agarose gel electrophoresis. Since it is difficult for LDLR^−/−^ mice to develop atherosclerosis by feeding on a chow diet only, we administered a HFD to the two groups of mice. After the mice were fed a HFD for 12 weeks, elastic fiber fracture was observed in Fbn1^C1039G+/−^LDLR^−/−^ mice, and this phenomenon was more obvious after 24 weeks of HFD, while elastic fibers in LDLR^−/−^ mice were unaffected. As shown in Figure 2, elastic fibers were intact and continuous in LDLR^−/−^ mice, whereas they exhibited progressive fragmentation in Fbn1^C1039G+/−^LDLR^−/−^ mice, which agrees with previous findings.

**Figure 3:**
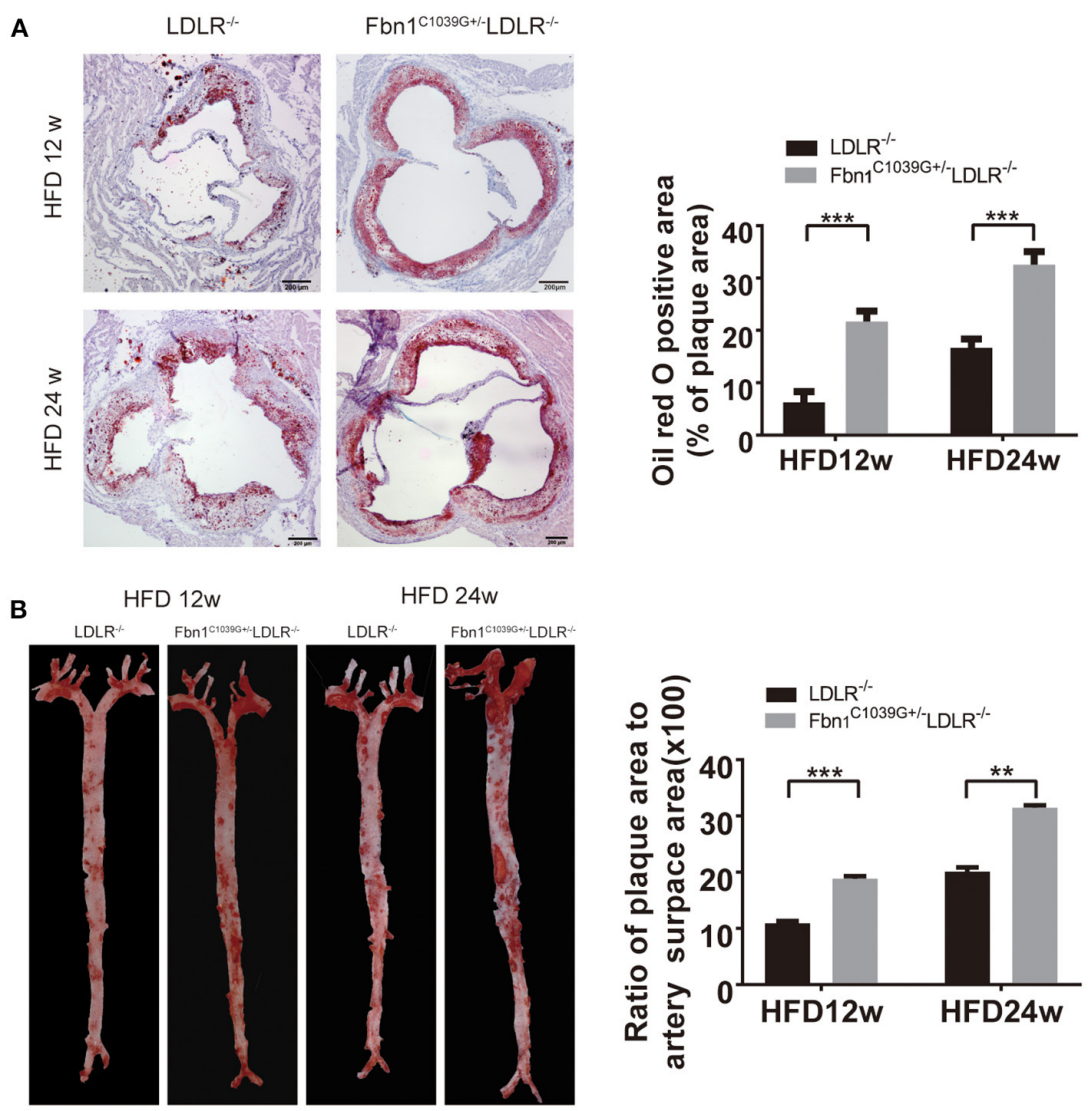
Pathological staining of the aorta and aortic sinus of Fbn1^C1039G+/−^LDLR^−/−^ and LDLR^−/−^ mice after 12 or 24 weeks on a HFD. **(A)** Oil red O staining of aortic sinus (*n* = 5 per group). **(B)** *En face* oil red O staining (*n* = 3 mice in each group). ^***^P < 0.001, ^**^P < 0.01. The results showed that the Fbn1^C1039G+/−^LDLR^−/−^ mice exhibited worsened atherosclerosis.

With the extension of time on the HFD, the degree of the atherosclerotic lesions in the two groups increased. Oil red O staining of the cross-sections of the aortic sinus and *en face* oil red O staining (the whole aorta from the aortic roots to the bifurcations of the iliac arteries) of Fbn1^C1039G+/−^LDLR^−/−^ and LDLR^−/−^ mice after 12 or 24 weeks of HFD are shown in Figures 3A,B. The results show that atherosclerotic lesions in Fbn1^C1039G+/−^ LDLR^−/−^ mice were significantly more severe than those in LDLR^−/−^ mice. More specifically, the positive area of oil red O staining in the aortic sinus of Fbn1^C1039G+/−^LDLR^−/−^ mice was significantly larger than that of LDLR^−/−^ mice after 12 and 24 weeks of HFD (Figure 3A). This phenomenon was also observed in plaques in the full-length aortas of Fbn1^C1039G+/−^LDLR^−/−^ mice compared with LDLR^−/−^ mice (Figure 3B).

### Identification of the Characteristics of the Plaque Phenotype in Fbn1^C1039G+/–^LDLR^–/–^ Mice

**Figure 4:**
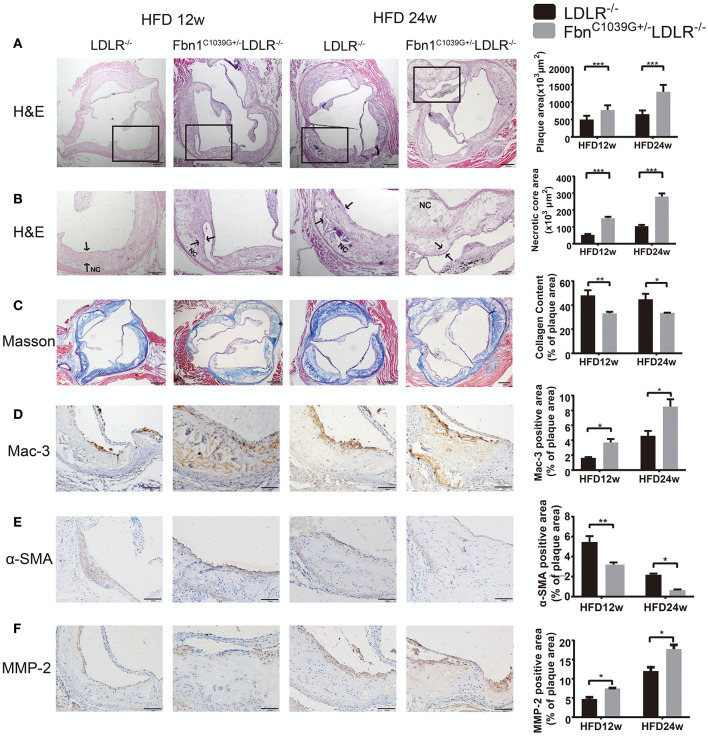
Plaque characteristics of Fbn1^C1039G+/−^LDLR^−/−^ and LDLR^−/−^ mice after 12 or 24 weeks on a HFD. **(A)** H and E staining of the aortic sinus of Fbn1^C1039G+/−^LDLR^−/−^ and LDLR^−/−^ mice after 12 and 24 weeks on a HFD (*n* = 9). **(B)** Partial enlargement of **(A)** showing the necrotic cores (*n* = 9). **(C)** Masson staining of the aortic sinus of the two groups of mice after 12 and 24 weeks on a HFD (*n* = 6). **(D)** Mac-3 staining of the aortic sinus of Fbn1^C1039G+/−^LDLR^−/−^ and LDLR^−/−^ mice after 12 and 24 weeks on a HFD (*n* = 4). **(E)** α-SMA staining of the aortic sinus of Fbn1^C1039G+/−^LDLR^−/−^ and LDLR^−/−^ mice after 12 and 24 weeks on a HFD (*n* = 4). **(F)** MMP-2 staining of the aortic sinus of Fbn1^C1039G+/−^LDLR^−/−^ and LDLR^−/−^ mice after 12 and 24 weeks on a HFD (*n* = 4). The arrows show the fibrous cap. NC, Necrotic core. ^***^P < 0.001, ^**^P < 0.01, ^*^P < 0.05.

To determine the characteristics of the plaque phenotype in Fbn1^C1039G+/−^LDLR^−/−^ mice, we performed a comprehensive evaluation of the atherosclerotic lesions of the mice. Cross-sectional analysis of the two groups of mice fed a HFD for 12 and 24 weeks was performed to identify the cellular composition and structural characteristics of atherosclerotic lesions. H&E staining of the aortic sinus was used to quantify atherosclerotic plaque size as well as the area of the necrotic core. The results showed that plaque size in the model mice was significantly higher than that in the control mice after 12 and 24 weeks of HFD feeding (Figure 4A). Accordingly, the area of the necrotic core was significantly larger and the fiber cap was thinner in the model mice than in the control mice. Statistical analysis showed that a necrotic core was one of the features of model mice, and the area was ~3 times that of the control mice (Figure 4B). Masson staining, which is a classic method to visualize collagen fibers, was used to compare the collagen content of atherosclerotic plaques between control and model mice. The results suggested that there were fewer collagen fibers in the model mice, and they were thin and sparsely distributed (Figure 4C). Additionally, compared to control mice, model mice fed a HFD showed abnormal vessel dilation with elastic fiber fragmentation in the arterial wall, and the blood vessel cross-sectional area was significantly larger (Table 1). Immunohistochemical staining of the aortic plaque was used to quantify macrophages, smooth muscle cells and MMP-2 content in plaques. Macrophage content was determined by calculating the percentage of the total plaque area that was positively stained for Mac3. As shown in Figure 4D, compared to that in control mice, the percentage of plaque area that was Mac3-positive was significantly increased in model mice after 12 and 24 weeks of HFD (Figure 4D). Smooth muscle cell content, expressed as the percentage of total plaque area that was positively stained for α-SMA, showed a marked decrease in model mice after both 12 and 24 weeks of HFD (Figure 4E). Immunohistochemistry also showed a significant rise in the percentage of MMP-2-positive plaque area in model mice (Figure 4F). Taken together, the results reveal that the characteristics of the plaque phenotype in Fbn1^C1039G+/−^LDLR^−/−^ mice are similar to those of human “vulnerable” plaques, including significantly increased vessel area, large plaque size and necrotic core, thin fibrous cap and decreased total collagen. The characteristics are more evident with increasing HFD time.

**Table 1:** Characteristics of Fbn1^C1039G+/−^ LDLR^−/−^ and LDLR^−/−^ mice after 12 or 24 weeks on HFD.

	**HFD 12 w**		**HFD 24 w**	
	**LDLR^**−/−**^**	**Fbn1^**C1039G+/–**^LDLR^**−/−**^**	**LDLR^**−/−**^**	**Fbn1^**C1039G+/–**^LDLR^**−/−**^**
Body weight (g)	22.3 ± 0.3	23.4 ± 0.4	23.8 ± 0.2^†^	24.6 ± 0.3
TC (mg/dl)	622 ± 61	635 ± 64	1,220 ± 107^†††^	1,134 ± 89^‡‡‡^
TG (mg/dL)	176 ± 9.9	177 ± 7.8	189 ± 5.2^†^	185 ± 7.9
HDL (mg/dL)	170 ± 15	181 ± 13	227 ± 26	230 ± 23
LDL (mg/dL)	233 ± 57	243 ± 37	734 ± 94^†††^	716 ± 51^‡‡‡^
Vessel area (10^3^ μm^2^)	1,416 ± 41	2,114 ± 46^***^	1,465 ± 49	3,010 ± 67^***^^‡‡‡^
miniFCT (μm)	44.8 ± 4.1	25.2 ± 3.5^**^	32.6 ± 2.7^††^	14.0 ± 2.8^**^^‡^
Rate of discontinued ECs	n.d.	n.d.	2/7	5/6^*^

*n.d., not determined*.

* *P < 0.05*,

** *P < 0.01*,

*** *P < 0.001 vs. the LDLR^−/−^ group*.

† *P < 0.05*,

†† *P < 0.01*,

††† *P < 0.001 vs. the LDLR^−/−^ group at 12 w HFD*.

‡ *P < 0.05*,

‡‡‡ *P < 0.001 vs. the Fbn1^C1039G+/−^ LDLR^−/−^ group at 12 w HFD*.

### Plaque Rupture and Vasa Vasorum Generation in Fbn1^C1039G+/–^LDLR^–/–^ Mice

**Figure 5:**
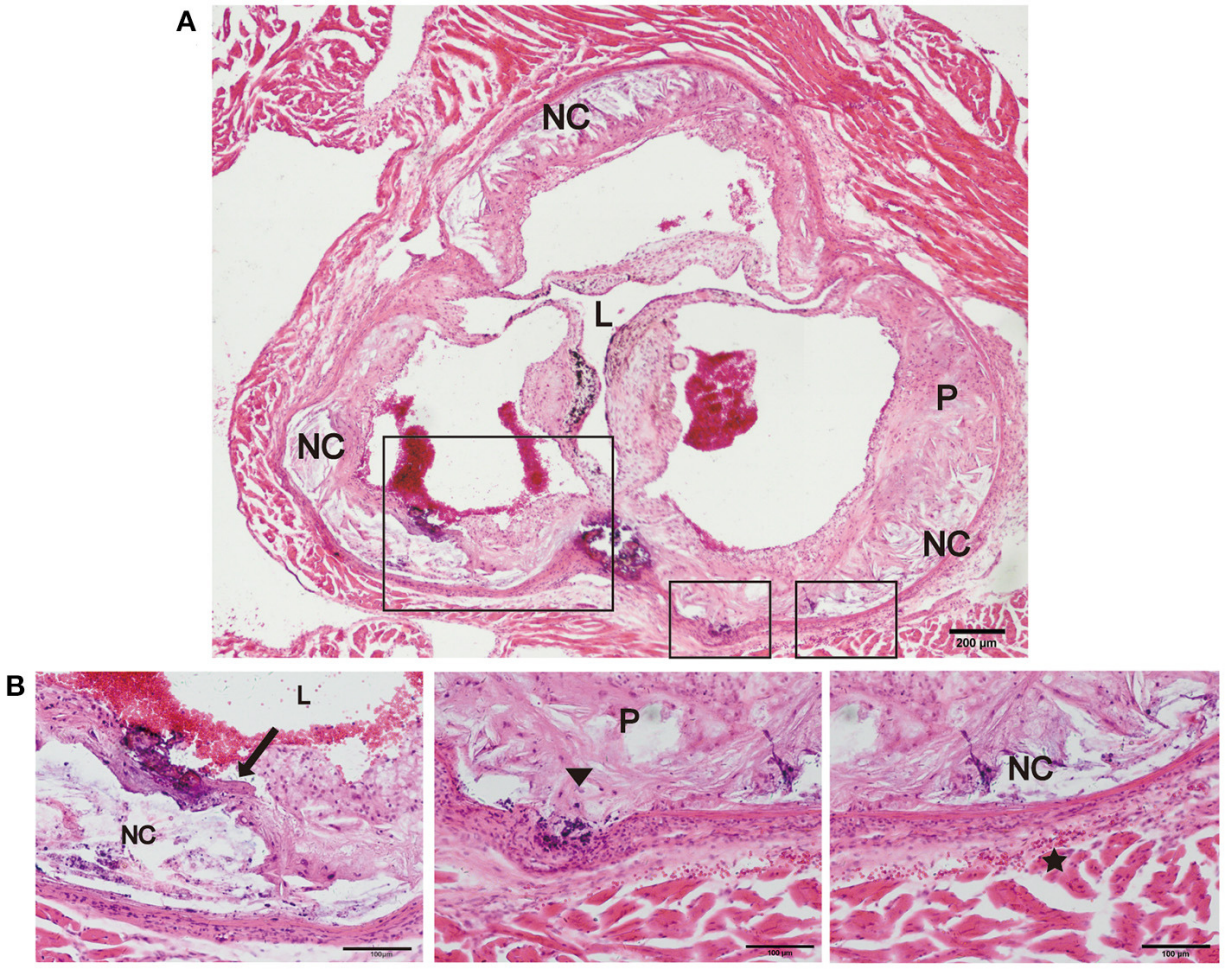
Plaque rupture and vasa vasorum generation in Fbn1^C1039G+/−^LDLR^−/−^ mice. **(A)** Plaque rupture and secondary thrombosis in Fbn1^C1039G+/−^LDLR^−/−^ mice after HFD feeding. **(B)** Partial enlargements of **(A)**. The arrow shows the ruptured fibrous cap, the triangle shows the fragmentation of elastin, and the star shows the vasa vasorum network. P, plaque; L, lumen; NC, necrotic core.

Previous studies have reported that an increase in arterial stiffness aggravated atherosclerotic plaque progression, and the plaques were vulnerable and prone to rupture (11, 16). In the present study, we focused on evaluating the vulnerability of atherosclerotic plaques in the new model. During HFD feeding for 12 and 24 weeks, there was no significant difference in body weight, and no neurological complications or acute cardiovascular events occurred in the two mouse models (Table 1). However, plaque rupture and thrombosis were observed in ~20% of the model mice fed a HFD (*n* = 10). In addition, we also found ruptured fibrous caps, elastic fiber fragments, and vasa vasorum in Fbn1^C1039G+/−^LDLR^−/−^ mice, indicating that these typical plaque characteristics bear a strong similarity to those of human advanced AS (Figures 5A,B).

### Damaged Endothelium, Intraplaque Hemorrhage, and Neovascularization in Fbn1^C1039G+/–^LDLR^–/–^ Mice

**Figure 6:**
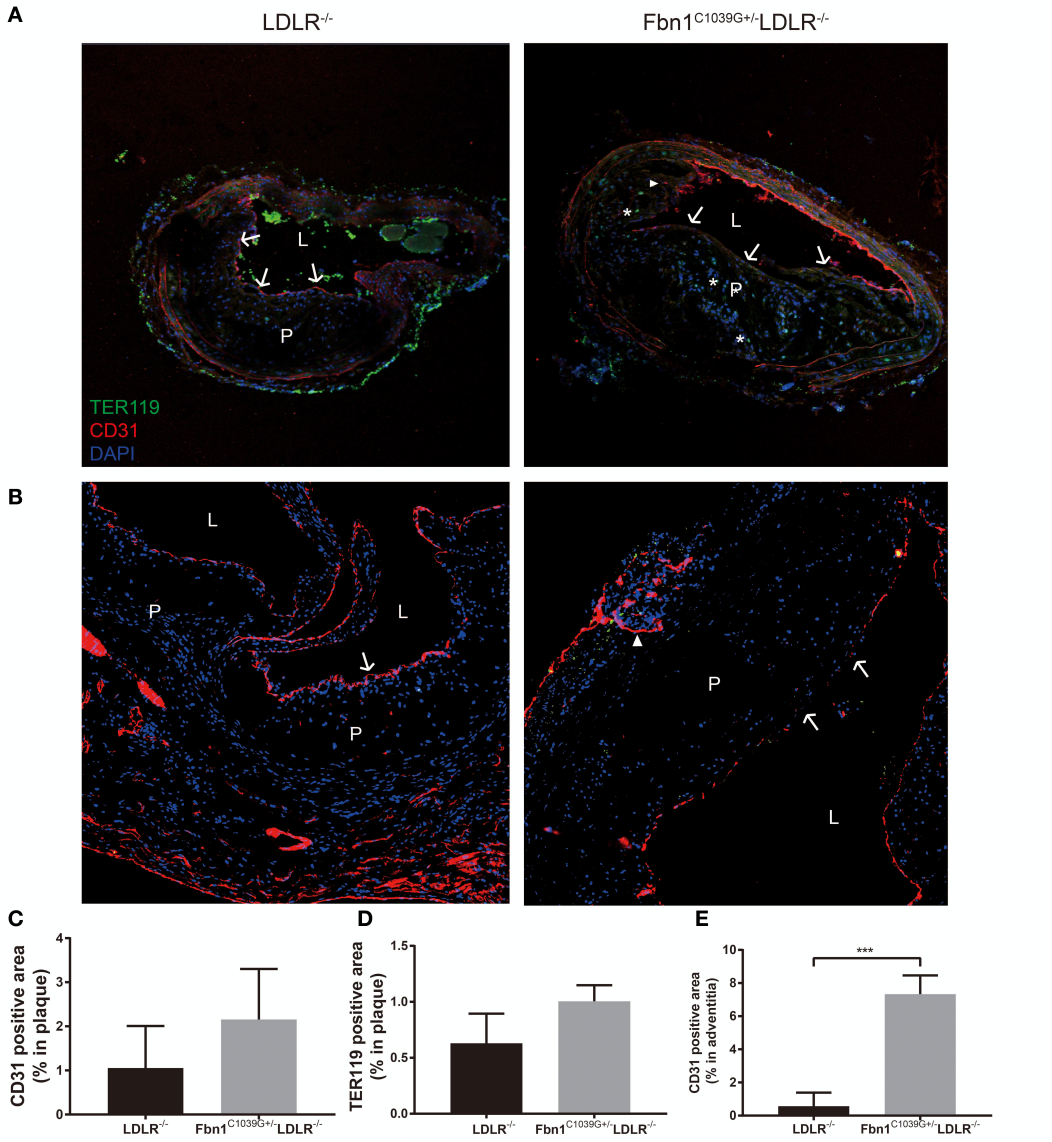
Damaged endothelium, intraplaque hemorrhage and neovascularization. **(A)** Immunofluorescence staining of the brachial artery in Fbn1^C1039G+/−^LDLR^−/−^ mice and LDLR^−/−^ mice after 24 weeks on a HFD. **(B)** Immunofluorescence staining of the aortic sinus in Fbn1^C1039G+/−^LDLR^−/−^ mice and LDLR^−/−^ mice after 24 weeks on a HFD. **(C)** Immunofluorescence staining quantification of the CD31-positive area (% of plaque) in Fbn1^C1039G+/−^LDLR^−/−^ mice and LDLR^−/−^ mice after 24 weeks on a HFD. **(D)** Immunofluorescence staining statistics of the TER119-positive area (% of plaque) in Fbn1^C1039G+/−^LDLR^−/−^ mice and LDLR^−/−^ mice after 24 weeks on a HFD. **(E)** Quantification of the CD31-positive area (% of media and adventitia) in Fbn1^C1039G+/−^LDLR^−/−^ mice and LDLR^−/−^ mice after 24 weeks on a HFD (*n* = 5 in Fbn1^C1039G+/−^LDLR^−/−^ mice, *n* = 3 in LDLR^−/−^ mice), ^***^p < 0.001. Magnification: ×100. TER119 shows red blood cells, CD31 shows endothelial cells, and DAPI shows nuclei. The arrow indicates the endothelial barrier, the triangle indicates neovascularization, and ^*^indicates intraplaque hemorrhage. P, plaque; L, lumen.

As barrier cells, endothelial cells have an important role in the occurrence and development of atherosclerosis. Endothelial dysfunction can lead to inflammatory cell invasion and lipid deposition in the subendothelial area, which can exacerbate atherosclerosis. With the increase in plaque size and the stenosis of the arterial lumen, haemodynamic changes often occur in vessels, exacerbating endothelial injury and promoting neovascularization (17). Due to the immature neovasculature, vessel walls are particularly vulnerable to rupture and leakage, resulting in intraplaque hemorrhage and plaque rupture (18). Because another prominent characteristic of human vulnerable plaques is the presence of intraplaque neovascularization, we further observed whether this feature was also present in our new model. Using immunofluorescence staining, the internal structural features of the plaques in the two models were observed after 24 weeks of HFD feeding. Compared to control mice, Fbn1^C1039G+/−^LDLR^−/−^ mice showed inner surface structural discontinuity and endothelial barrier impairment, as shown in Figures 6A,B. Furthermore, neovascularization and intraplaque hemorrhage were detected in model mice, but no signs of neovascularization or intraplaque hemorrhage were observed in control mice (Figures 6A,B). According to statistical analysis, there was no difference in the percentages of TER119- or CD31-positive plaque areas in control and model mice (Figures 6C,D). However, there was a significant difference in the percentage of TER119-positive areas in the adventitia and media between the two groups (Figure 6E).

## Discussion

ACS is known to be a major cause of death from coronary heart disease (CHD) that has a high disability rate and poor prognosis. Although it has been demonstrated that the existing prevention and treatment strategies have a certain therapeutic effect against CHD, the incidence of ACS and complications caused by VP rupture is still high and has become an urgent clinical problem to be solved. With the development of basic medicine, the pathophysiological mechanism of CHD has been extensively studied. However, due to the lack of an ideal animal model for VP, it is currently difficult to transform preliminary basic research into clinical applications. Arterial stiffness is caused by aging or risk factors and mainly manifests as the degradation of extracellular matrix (ECM), which is characterized by collagen deposition and elastic fiber fracture and is a degenerative process of elastic arteries. Research has found that in terms of pathophysiology, arteriosclerosis and AS are synergistic processes that potentiate each other to exacerbate vascular damage (19). On the one hand, in stiff arteries, diastolic blood pressure (DBP) falls sharply, leading to insufficient diastolic coronary perfusion, which may exacerbate myocardial ischaemia under AS (20). On the other hand, the development and progression of AS, especially plaques, can promote the destruction of elastic fibers and outward vascular remodeling, resulting in the enlargement of the luminal area and worsening the severity of arterial stiffness (21).

Fibrillin is the primary component of microfibrils and is encoded by the Fbn1 gene. Mutation of the Fbn1 gene impairs elastic fiber cross-linking, reduces the elasticity of vessels, and affects the structure of the aortic wall. In the cardiovascular system, rupture of elastic fibers can cause arteriosclerosis and dilation of the ascending aorta, which may complicate aortic dissection or aneurysm (11). In this study, we introduced a fibrillin-1 mutation into LDLR^−/−^ mice via homologous recombination to construct a novel AS model. After feeding the mice a HFD for 12 or 24 weeks, the characteristics of the model were evaluated using multiple pathological experiments. The results showed that elastic fiber fracture was observed in Fbn1^C1039G+/−^LDLR^−/−^ mice (Figure 2). Although Fbn1^C1039G+/−^LDLR^−/−^ mice showed no significant difference in lipid level compared with LDLR^−/−^ mice, the positive areas of oil red O staining in the aortic sinus and full-length aorta increased significantly (Figures 3A,B). The results showed that atherosclerotic lesions of Fbn1^C1039G+/−^LDLR^−/−^ mice were more severe than those of LDLR^−/−^ mice, indicating that arteriosclerosis with fiber fracture can exacerbate the development of AS. Further analysis of the plaque phenotype established that the plaques of model mice were more unstable and had characteristics similar to those of human VP. Fbn1^C1039G+/−^LDLR^−/−^ mice fed a HFD were observed to have larger necrotic cores, thinner fibrous caps, more inflammatory cells, increased MMP-2, and significantly fewer collagen fibers and SMCs than control mice (Figures 4A–F). A thin fibrous cap mainly consisting of SMCs, ECM and collagen fibers is recognized as a precursor to atherosclerotic plaque rupture. In the process of AS development, with endothelial dysfunction and SMC phenotype switching, SMCs proliferate and migrate around the fibrous cap to stabilize the plaque. In turn, the SMCs in the fibrous cap migrate into the plaque core. Then, due to stimulation by inflammatory factors, many SMCs undergo apoptosis, resulting in a decrease in SMC content (Figure 4E) and thinning of fiber caps, increasing the likelihood of plaque rupture (22, 23). Therefore, in the present study, the thinning of the fiber cap in the model mice might be associated with the surge of inflammatory factors and the apoptosis of SMCs. Further experiments are still needed to explore the specific mechanism.

Moreover, compared to that of LDLR^−/−^ mice, the percentage of Mac3-positive area was significantly increased in the plaques of Fbn1^C1039G+/−^LDLR^−/−^ mice after 12 and 24 weeks of HFD (Figure 4D), and the total number of macrophages was closely related to the progression and severity of plaques. It is well-known that macrophage accumulation in vessel walls is a hallmark of the development of atherosclerosis (24, 25). Stimulated by multiple environmental factors, macrophages can switch to many kinds of functional phenotypes that play essential roles in plaque phenotype and stability (26, 27). Studies have revealed that M1 macrophages are mainly located in unstable areas of plaques and are associated with plaque progression. In contrast, M2 polarization markers are mainly expressed in the stable zones and are associated with plaque regression (26, 28). Proinflammatory M1 macrophages can secrete multiple inflammatory cytokines, such as TNF, IL-6, and IL-1β, which activate endothelial cells, promote SMC proliferation, reduce collagen synthesis and matrix degradation and finally aggravate VP progression (24, 29). Matrix metalloproteinases (MMPs), which are widely produced by monocytes/macrophages, SMCs, endothelial cells, fibroblasts, and neoplastic cells, play a crucial role in protein degradation (30). Studies have demonstrated that MMP dysregulation may be involved in vascular and cardiac remodeling and atherogenesis (31, 32). In addition, similar to M1 macrophages, MMPs are highly expressed in unstable areas of atherosclerotic plaques and may have the ability to promote the destabilization of plaque lesions (33). In this study, we detected the content of MMP-2 in plaques and observed a significant increase in model mice (Figure 4F); this may be an important factor leading to plaque instability. However, more work is needed to observe the spatial distribution of different phenotypic macrophages and the regulatory effect of MMPs on atherosclerotic lesions in model mice.

The LDL receptor is a membrane receptor that mediates the plasma level of cholesterol-rich LDL. Studies have found that LDLR deficiency significantly affects the uptake and clearance of LDL as well as other lipoproteins, resulting in abnormal plasma lipid metabolism (34, 35). Pathological studies have demonstrated that LDLR^−/−^ mice fed with HFD have been widely used as an atherosclerotic model for basic research. Nevertheless, LDLR^−/−^ murine models develop relatively less severe coronary atherosclerosis but require a long-term HFD, which places certain limitations on mechanistic research of VP (36). Apolipoprotein E (ApoE) is a lipoprotein that exerts antiatherogenic effects by affecting lipoprotein metabolism directly and indirectly during the development of atherosclerosis. ApoE is a high-affinity ligand of a majority of lipoprotein particles, including LDL-, LDLR- and LDLR-related proteins, and plays an important role in lipoprotein remnant clearance (37). ApoE^−/−^ mice spontaneously develop hyperlipidaemia and extensive atherosclerosis even on a normal chow diet. However, neither ApoE^−/−^ nor LDLR^−/−^ mice can fully represent the characteristics of atherosclerotic plaque rupture in humans. Van der Donckt et al. found that mice with the Fbn1^C1039G+/−^ mutation show a series of pathophysiological changes, such as elevated pulse pressure, progressive aortic dilation and decreased contractility of cardiomyocytes, resulting in arterial stiffness and cardiac dysfunction (11). ApoE^−/−^Fbn1^C1039G+/−^ mice can be a perfect model of human end-stage atherosclerosis for exploring the specific mechanism of plaque destabilization and therapeutic targets for advanced atherosclerosis (38). However, sudden death and typical neurological complications can be observed in ApoE^−/−^Fbn1^C1039G+/−^ mice during HFD. Moreover, there was a 50% mortality rate after 20 weeks of HFD (39). In the present study, during the whole period of high-fat diet feeding, all of the mice were in good condition, and no neurological complications or acute cardiovascular events were recorded, which is in contrast to a previous study using ApoE^−/−^Fbn1^C1039G+/−^ mice (11). Asymptomatic plaque rupture of this model mouse might be related to less vascular stenosis and lesion thrombosis (40). This may also have occurred because the infarction vessel was not located in an important position; thus, no corresponding symptoms occurred. These hypotheses need to be verified by further pathological tests of the heart and brain. This characteristic enables the model mice to avoid the problem of high mortality observed in ApoE^−/−^ Fbn1^C1039G+/−^ mice and may reduce the interference of high mortality in subsequent studies.

Furthermore, elastic fiber fragments, plaque rupture, and thrombosis were observed in Fbn1^C1039G+/−^LDLR^−/−^ mice, indicating a highly unstable plaque phenotype that bears a strong similarity to that of human advanced atherosclerosis (Figures 5A,B). In addition, compared with LDLR^−/−^ mice, inner surface structural discontinuity and endothelial barrier impairment appeared in Fbn1^C1039G+/−^LDLR^−/−^ mice. Neovascularization and intraplaque hemorrhage were observed within the plaque regions of the model mice (Figure 6). Neovascularization and intraplaque hemorrhage are prominent characteristics of human vulnerable plaques and are closely related to future ischaemic events (41). In general, the vasa vasorum is generated when the vasculature is damaged and provides oxygen and nutrients for tissue repair (42). However, the vasa vasorum, influenced by lipid deposition, endothelial injury, and a hypoxic inflammatory microenvironment, plays an intricate role in the progression of plaques (43). With progressive intimal thickening and luminal narrowing, the hypoxic area further expands, causing vasa vasorum to grow across the vessel wall to the lumen, thereby supplying oxygen and nutrients to the inner layers (44–46). Since ~80% of the new vessels are immature, vessel walls are particularly vulnerable and easily rupture and leak, leading to intraplaque hemorrhage and plaque breakage (18). Erythrocyte exudation can exacerbate the local inflammatory response, accelerating the progression of plaques (45).

Therefore, as a VP mouse model with spontaneous plaque rupture, this model is of great significance to drug development, equipment upgrading, and further exploration of the mechanism of atherosclerosis. Moreover, compared with pig or dog models, mouse models have a shorter breeding cycle, lower feeding costs and are easier to use. Compared to models built by balloon injury or artery ligation, this model better simulates the pathological physiological process of human vulnerable plaques. In addition, mouse models with the C57BL/6 background have high repeatability, which is beneficial to researchers exploring the characteristics and intervention means of vulnerable plaques.

However, there are still some limitations in our study: (1) we neglected to compare the liver conditions, such as inflammation, fibrosis or steatosis, of the two groups; (2) only a small number of mice were used for some staining experiments; and (3) additional methods, such as RT-PCR or WB, should be used to evaluate plaque vulnerability. Future study to address these problems is needed.

## Conclusion

In conclusion, the Fbn1^C1039G+/−^LDLR^−/−^ mouse is a suitable model for the study of vulnerable plaques. Compared to the LDLR^−/−^ mice, the model mice exhibited a prominent increase in plaque destabilization. The comprehensive analysis revealed that the plaques in the model had the following characteristics of vulnerable plaques: (1) larger necrotic cores, thinner fibrous caps, and significantly fewer collagen fibers and smooth muscle cells; (2) more inflammatory cells and MMP-2 content; (3) spontaneous plaque rupture and secondary thrombosis; and (4) neovascularization and intraplaque hemorrhage.

## Data Availability Statement

The raw data supporting the conclusions of this article will be made available by the authors, without undue reservation.

## Ethics Statement

The animal study was reviewed and approved by Ethics Committee of the Second Affiliated Hospital of Harbin Medical University.

## Author Contributions

XW and YF performed the animal experiments and wrote the paper. ZX and MC raised the animals and collected the tissues. WQ and XX conducted the pathological experiments. SZ and MP designed the primers and conducted the PCR assays. XP and YJ analyzed the data. LM and JT conceived of, designed, and supervised the study. All authors read and approved the final manuscript.

## Conflict of Interest

The authors declare that the research was conducted in the absence of any commercial or financial relationships that could be construed as a potential conflict of interest.

## Abbreviations

ACS: acute coronary syndrome
VP: vulnerable plaque
HFD: high-fat diet
AS: atherosclerosis
SMC: smooth muscle cells
Fbn1: Fibrillin1
ApoE^−/−^: apoprotein E–deficient mice
LDLR^−/−^: low-density lipoprotein (LDL) receptor-deficient mice
H&E: Haematoxylin-eosin
CHD: coronary heart disease
ECM: extracellular matrix
DBP: diastolic blood pressure
MMP: matrix metalloproteinase.
